# Correlation between Squamous Suture and Sylvian Fissure: Osirix DICOM Viewer Study

**DOI:** 10.1371/journal.pone.0018199

**Published:** 2011-03-31

**Authors:** Nunung Nur Rahmah, Takahiro Murata, Takehiro Yako, Tetsuyoshi Horiuchi, Kazuhiro Hongo

**Affiliations:** 1 Department of Neurosurgery, Shinshu University, Matsumoto, Japan; 2 Department of Neurosurgery, Aizawa Hospital, Matsumoto, Japan; The University of Chicago, United States of America

## Abstract

**Background:**

Sylvian fissure (SF) is an important corridor in neurosurgery, and the end of sylvian fissure (eSF) represents the optimal target area to expose suitable recipient artery in STA-MCA bypass. Unfortunately little have been addressed concerning its relationship with external cranial surface.

**Objective:**

Correlation between Squamous Suture (SS) and SF was investigated.

**Methods:**

50-adult 3D-CTA images were studied using OSIRIX DICOM viewer. The measurement points were determined from external auditory meatus 0, 1, 1.5, 2, 2.5, 3, 3.5 and 4-cm anteriorly, perpendicular from orbitomeatal (OM) line. The distance of SF was compared with the one of SS.

**Results:**

SSs were all located below SF at 0 cm. At a distance of 0 to 1.5 cm, SSs were located above SF, then started to merge and went side by side from 2 cm anteriorly. Anterior sylvian point, the most anterior part of SF, was found at 4 cm from OM line. Inferior Rolandic point, which corresponds to the central sulcus inferior extent, was found to be at 2 cm from OM line. The eSF was identified at 0 cm anteriorly from OM, and perpendicularly 1.5 cm above SS. 50% patients had Chater's point (CP) above eSF. Average value for CP was 0.01 below eSF, giving a significantly closer value compared to the one of SS (p<0.01). However, SS showed consistent value of 1.5 below SF. Furthermore, SS is a bony landmark, which has no shifting effect during surgery, therefore drawing a 1.5-cm line upward from SS could lead to exact location of eSF.

**Conclusion:**

The course of SF and its correlation to SS have been identified, and this is also the first study to investigate the relationship of SS and eSF using OSIRIX DICOM viewer. SS is also comparable to CP, therefore it is usable for a simple landmark of eSF.

## Introduction

Pterional approach, which has become the basic surgical approach for most neurosurgeons, was first introduced by Yasargil in 1969.[1] It is now widely used in operations for tumors of orbital, retroorbital, sellar, subfrontral, prepontine areas, medial sphenoid ridge, the superior orbital fissure, anterotemporal surface, or the cavernous sinus region. It is also important for operations of anterior circulation and upper basilar artery aneurysms. The sylvian fissure (SF) then became the microneurosurgical corridor to the base of the brain. Careful technique of its dissection is the real key to successful operations, and well-planned surgical orientation is a prerequisite. Yasargil and other authors have mentioned detailed descriptions of the SF anatomy and opening technique,[2]–[7] however, little attentions have been addressed concerning its relationship with the external cranial surface.

Ribas et al.[8] studied the correlation of anterior sylvian point and external cranial surface. They found out that the anterior sylvian point was located underneath the 1.5-cm-diameter cranial area of the anterior aspect of the squamous suture (SS). Gibo et al. in their study of microsurgical anatomy of the middle cerebral artery mentioned a distance from external auditory meatus (EAM) to anterior part of the SF was 47 mm.[9] However, those studies were cadaveric studies and no studies have mentioned in details the correlation of SF and SS.

In this study, we are aiming at finding out the actual correlation between the two from patient's images. SS can be easily observed during surgery, therefore using SS as a surface landmark for SF will give advantages to surgeons without the need of using expensive technology.

## Materials and Methods

### Ethics statement

This study was approved by the local Ethical Committee of Shinshu University School of Medicine under registration number of 1359 and written informed consent was obtained from all the patients who participated in the clinical trials.

Fifty consecutive adult patients from Shinshu University Hospital who underwent three-dimensional computed tomography angiography (3D-CTA) and showed no mass-effect intracranial lesion were included in the study. The study group included 25 men and 25 women with a mean age of 59 years (range 22–82 years).

Images were acquired on a General Electric 64-row scanner (GE Yokogawa Medical systems, Tokyo, Japan). Scan parameters were 120 kv, auto setting of electric current of about 350 mA, individual detector collimation 64×0.625 mm, 40-mm detector length, gantry rotation time of 0.40 s, slice thickness of 1.25 mm or less and space between slices of 0.625 mm. Our routine protocol for angiographic study was performed, which included an arterial phase contrasted by 100–110 ml of Iopamiron®iopamidol (a triiodinated, non-ionic contrast agent, in 3.7 mg ml^−1^ concentration), administered intravenously approximately in 25 seconds, was adjusted automatically for every patients. Images were stored in compact discs and were saved as Digital Imaging and Communications in Medicine (DICOM) files.

**Figure 1:**
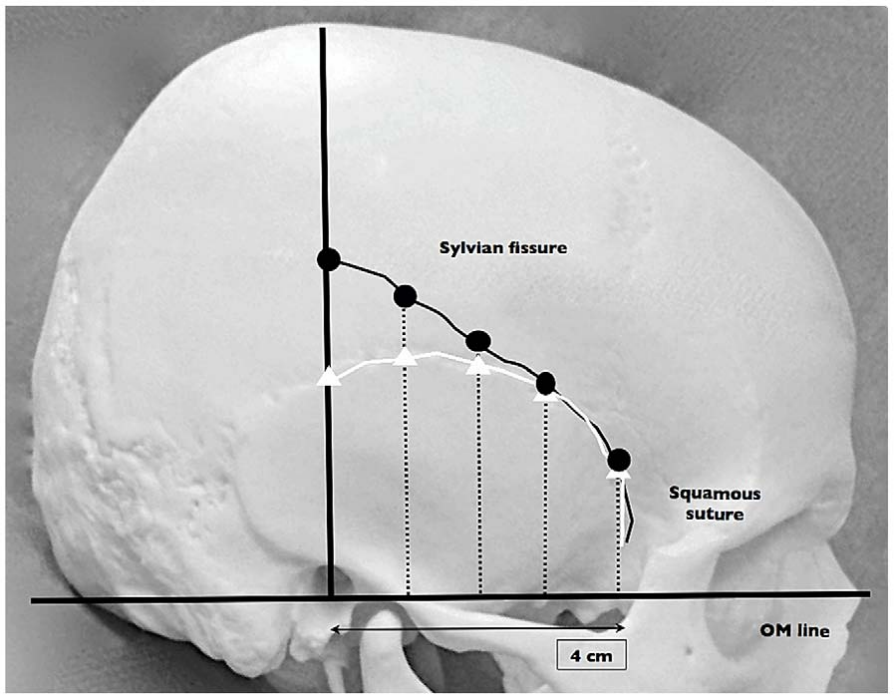
Illustrative methods of measurement. Orbitomeatal (OM) line is drawn to define the base of measurement. Perpendicular line to EAM is drawn, and is regarded as horizontal 0 cm. Curved-black line is showing SF. White line is SS. Dotted lines are showing the measurement points, from 0 to 4 cm anteriorly.

Osirix 3.3.2-DICOM viewer for Mac OS X was used. Images were viewed using two-dimensional (2D) multi planar reconstruction (MPR) with sagittal view as the center of orientation. Horizontal and vertical lines were adjusted until the standard orbito meatal (OM) plane was observed; it was defined as the plane between the largest lens seen and EAM (Figure 1). Balance between axial, coronal and sagittal view was adjusted by examiner. To demonstrate clearly SS and SF, image brightness was adjusted using the available tool by dragging the mouse around the image. By using the sagittal view as the orientation, blue horizontal line and red vertical line were seen. It was rotatable 360° on its arm, and could be moved vertically or horizontally on its center. The horizontal line was placed along the OM line, and the vertical line was placed perpendicular to OM line just on the EAM.

**Figure 2:**
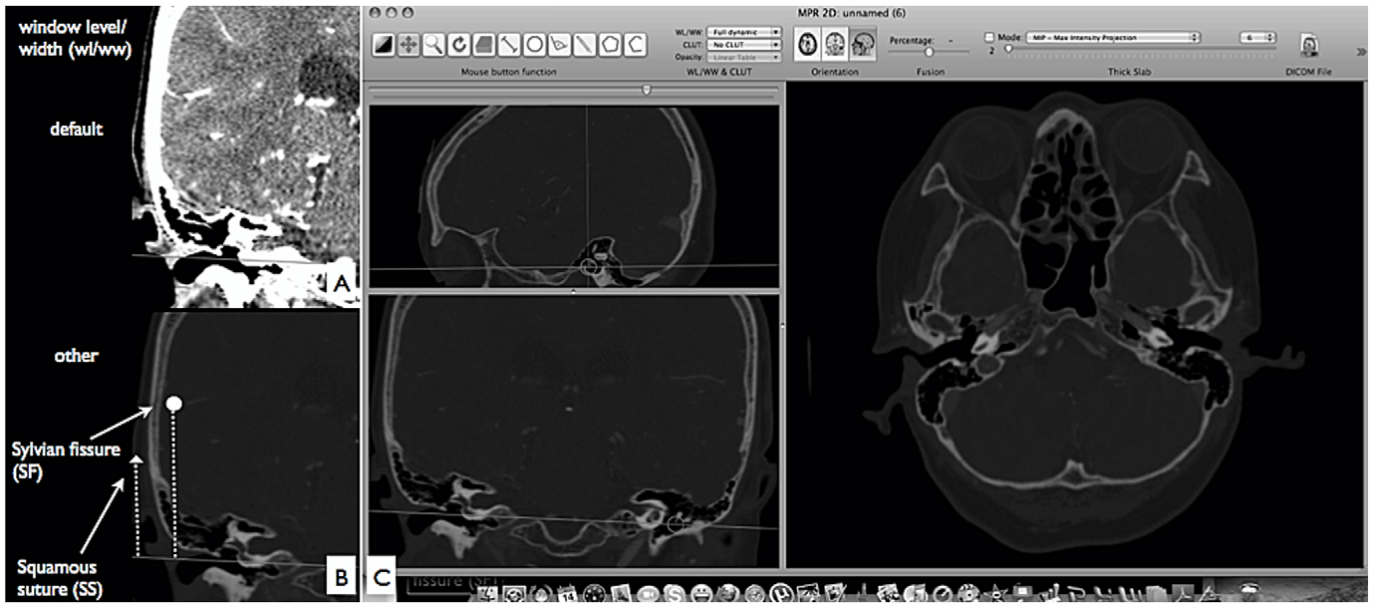
Interface of Osirix DICOM viewer for Sylvian fissure course analysis. a) Coronal view of default window/width level, which can show better visualization of the brain and vessels. b) Coronal bone window view, which can show a better visualization of the SS. White triangle is showing the length of SS, and white dot is showing the length of SF. c) Overall view of 2-D MPR; Crossed lines on sagittal image are movable to desired locations.

The length of SS was created by drawing a perpendicular line from OM to the point of SS observed on coronal view. The length of SF was also created in a similar fashion. The length of both SS and SF was measured at 0, 1, 1.5, 2, 2.5, 3, 3.5, and 4 cm from EAM, respectively. Region of interest (ROI) was saved as distance value (in centimeter). Measurement was performed on right and left sides (Figure 2).

**Figure 3:**
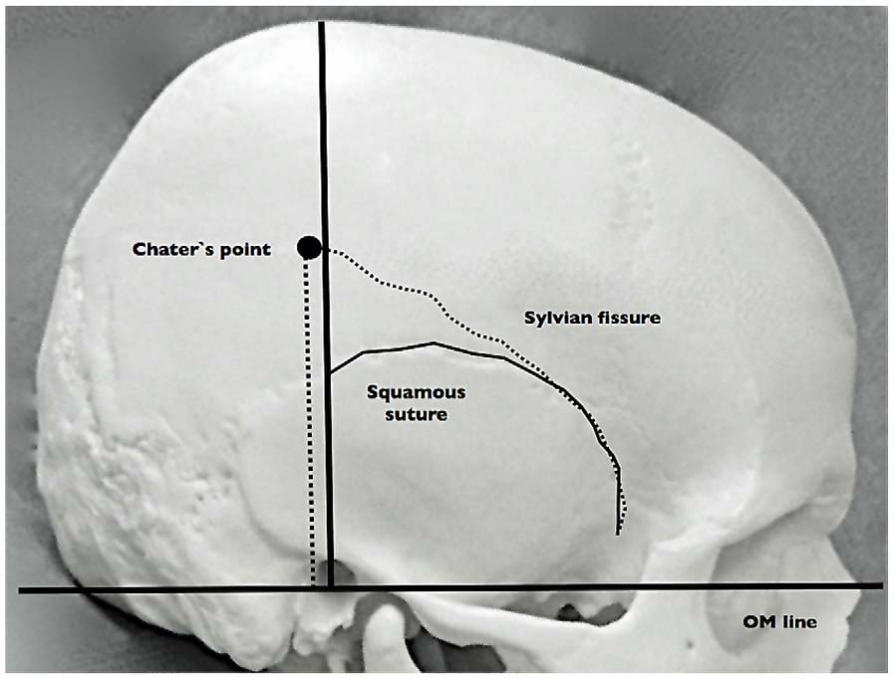
Illustrative methods of comparative measurement of Chater's point and Squamous suture. Orbitomeatal (OM) line is drawn to define the base of measurement. Perpendicular line to EAM is drawn, and is regarded as horizontal 0 cm. Small-dotted line is showing the sylvian fissure. Curved-black line is SS. Bigger dotted line is showing the presumed position of CP, 6 cm from EAM.

Another aspect that we would like to find out is comparison between our method using SS and the standard Chater's point (CP). In 1976, Chater et al. mentioned a point of 6 cm above the EAM perpendicular to skull base line as a surface landmark for the end of sylvian fissure (eSF), which was used for identifying the vessels around angular gyrus in bypass surgery.[9] [10]. CP was then created by drawing 6 cm perpendicular line on coronal view which synchronized the position generated on sagittal view. A point on the bone was connected to a point on SF, and region of interest (ROI) was saved as distance value (in centimeter). Another line for comparison was drawn from SS seen on the same coronal view to the exact point on SF measured before (Figure 3). Again, ROI was saved and used for analysis later.

### Statistical Analysis

Statistical analysis was performed using SPSS 14 for windows. T test was used to compare means between two independent variables. The level of significance (p) was set at a probability value of less than 0.05.

## Results

### 3D-CTA-based data

Patients who underwent the 3D-CTA examination were mostly aneurismal patients (33), followed by infarctions, cerebral arteriovenous malformation, tumors, and subarachnoid hemorrhage (SAH; 6, 4, 4, and 3 patients, respectively). No significant mass effect was found in the series. Patients with obvious widening of SF (caused by age-related atrophy, tumor or SAH) were excluded. 3 SAH patients in our series were minor SAH and they did not cause obvious changes of SF size. All patients' data in a form of DICOM files were easily opened using Osirix DICOM viewer. SS and SF could be viewed in all 50 patients.

On the right side, SSs were all located below SF at 0 cm measurement. At 1, 1.5, 2, 2.5, 3, 3.5, and 4 cm, SS in 4 (8%), 9 (18%), 19 (38%), 21 (42%), 30 (60%), 26 (52%), and 30 (60%) cases were located above SF, respectively (Table 1). At a distance of 0, 1 and 1.5 cm, SS and SF had significantly different means (p<0.01, 0.01, 0.01, respectively). However, mean value at a distance of 2 cm to 4 cm did not show any difference (Table 2).

**Table 1:** Mean value of length of Squamous suture and Sylvian fissure in 50 patients (Right).

Category	Distance from external auditory meatus (anteriorly)							
	0 cm	1 cm	1.5 cm	2 cm	2.5 cm	3 cm	3.5 cm	4 cm
Mean SS	4.48	4.66	4.71	4.71	4.58	4.40	4.10	3.79
Mean SF	6.00	5.59	5.22	4.88	4.58	4.30	4.02	3.71
SS above SF	0 (0%)	4 (8%)	9 (18%)	19 (38%)	21 (42%)	30 (60%)	26 (52%)	30 (60%)
SS below SF	50 (100%)	46 (92%)	41 (82%)	31 (62%)	29 (58%)	20 (40%)	24 (48%)	20 (40%)

SS: Squamous suture, SF: Sylvian fissure.

**Table 2:** P value for Squamous suture and Sylvian fissure in each location measured (Right).

Distance from EAM (cm)	Measurement		P value
	SS	SF	
0	4.48	6.00	**<0.01**
1	4.66	5.59	**<0.01**
1.5	4.71	5.22	**<0.01**
2	4.71	4.88	0.097
2.5	4.58	4.58	0.824
3	4.40	4.30	0.864
3.5	4.10	4.02	0.64
4	3.79	3.71	0.586

**Figure 4:**
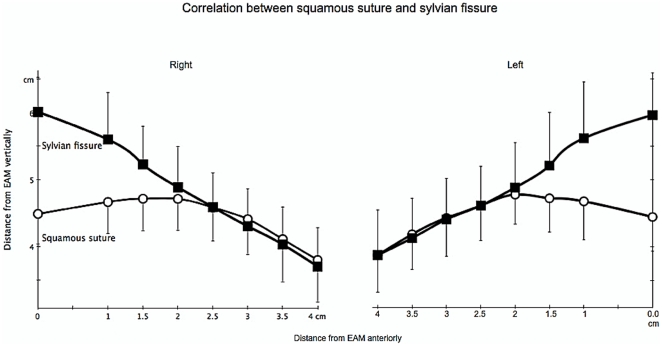
Correlation between Squamous suture and Sylvian fissure.

On the left side, the value was quite similar. At 0 cm, SSs were all located below the SF. At 1, 1.5, 2, 2.5, 3, 3.5, and 4 cm, SS in 6 (12%), 9 (18%), 25 (50%), 28 (56%), 29 (58%), 32 (64%), and 28 (56%) cases were located above SF, respectively (Table 3). At a distance of 0, 1 and 1.5 cm, SS and SF had significantly different means (p<0.01, 0.01, and 0.01 respectively). However, mean value at a distance of 2 to 4 cm did not show any difference (Table 4). Detailed correlation between SS and the SF is described in Figure 4.

**Table 3:** Mean value of length of Squamous suture and Sylvian fissure in 50 patients (Left).

Category	Distance from external auditory meatus (anteriorly)							
	0 cm	1 cm	1.5 cm	2 cm	2.5 cm	3 cm	3.5 cm	4 cm
Mean SS	4.44	4.67	4.72	4.77	4.61	4.43	4.18	3.88
Mean SF	5.96	5.61	5.21	4.88	4.61	4.40	4.13	3.88
SS above SF	0 (0%)	6 (12%)	9 (18%)	25 (50%)	28 (56%)	29 (58%)	32 (64%)	28 (56%)
SS below SF	50 (100%)	44 (88%)	41 (82%)	25 (50%)	22 (44%)	21 (42%)	18 (36%)	22 (44%)

SS: Squamous suture, SF: Sylvian fissure.

**Table 4:** P value for Squamous suture and Sylvian fissure in each location measured (Left).

Distance from EAM (cm)	Measurement		P value
	SS	SF	
0	4.44	5.96	**<0.01**
1	4.67	5.61	**<0.01**
1.5	4.72	5.21	**<0.01**
2	4.77	4.88	0.312
2.5	4.61	4.61	0.897
3	4.43	4.40	0.880
3.5	4.18	4.13	0.757
4	3.88	3.88	0.885

**Figure 5:**
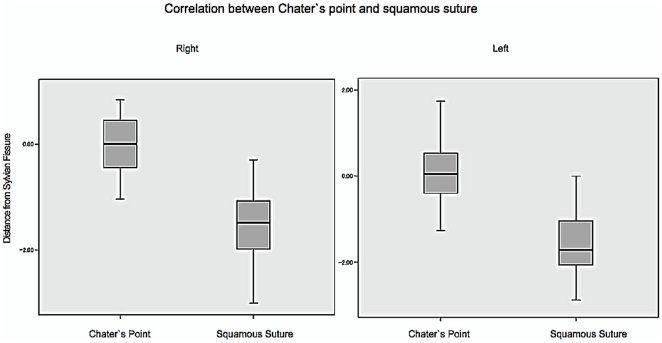
Correlation between Chater's point and Squamous suture.

Distance of SS and the standard CP from SF showed interesting results. On the right side, SSs were all located below SF in 50 patients, with an average of 1.52 cm below the SF at the EAM level perpendicular to OM line. 25 (50%) patients had CP above SF. Average value for CP was 0.01 below SF, which was significantly closer compared to SS on the same coronal plane (p<0.01). The value was similar to the left side. SSs were all located below SF, averaged 1.51 cm. 24 patients showed CP above SF, and 26 patients showed the opposite. CP was also significantly closer to SF compared with SS on the left side (p<0.01) (Figure 5).

### Application in actual bypass surgery

**Figure 6:**
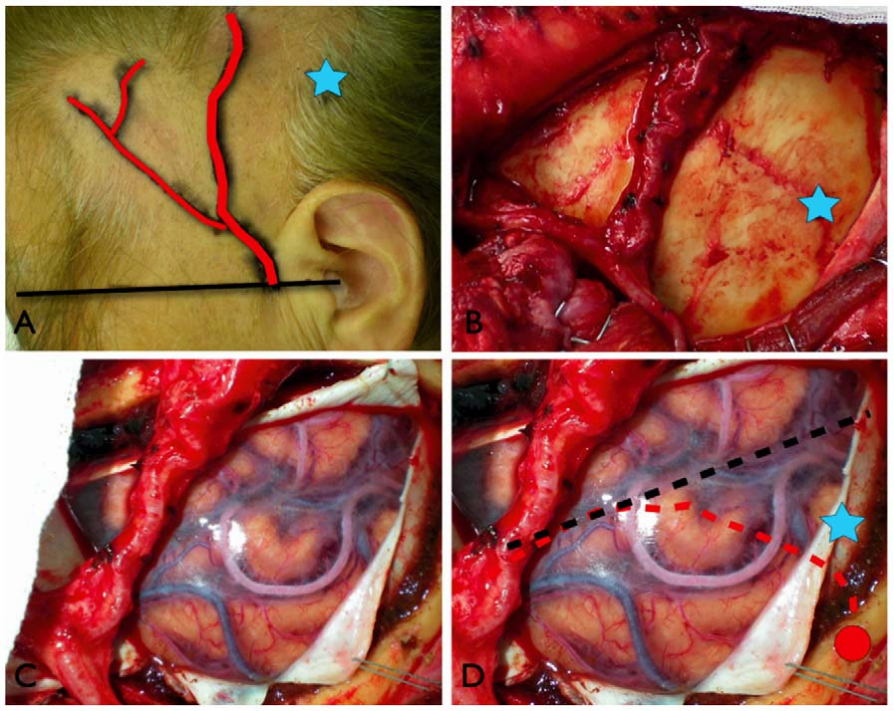
Clinical application of Squamous suture landmark in a representative case. a) Skin marking of the OM line (black line), the donor artery (STA, red line) and Chater's Point (CP, blue star). b) Bone marking using SS as the center of the craniotomy, the adjacent STA is also visible; blue star is showing the CP. c) The clearly viewed SF with its correlated arties and veins at the center of the operative field after craniotomy. d) Illustration of the running SS (dotted red line), SF (dotted black line), CP (blue star) and start point of SS at 0 cm perpendicular to OM line (red circle).

It is a preliminary result; however, for the period of one year in 2010 we have performed STA-MCA anastomosis using the described technique in 5 patients, 4 women and 1 man. Average age of the patients was 40.6 (13–68) years; 3 patients suffered from moya-moya disease and 2 patients were with internal carotid artery stenosis. All patients underwent superficial temporal artery-middle cerebral artery (STA-MCA) bypass surgery. SS was easily identified in all patients during the surgical procedure, and eSF was successfully identified using SS as landmark. Effective craniotomy could be achieved, all bypass results were patent, and there were no surgery-related complications postoperatively. One representative case is described in Figure 6.

## Discussion

### Usage of Squamous suture for identification of the end of Sylvian fissure (eSF)

eSF has been used widely to find the suitable donor artery in STA-MCA bypass. Several techniques to point the exact location have been employed, ranging from superficial landmarks to image-guided landmark.[9], [11]–[19] First skin-marking study was described in 1976 by Norman Chater et al., in their microvascular bypass surgery study aiming to find the best location to obtain adequate-size vessel. The point was said to be 6 cm above the EAM perpendicular to skull base line.[9]–[19] It has been a standard use ever since. However, the study was done in 1976 and the method was based on cadaveric study using skin marking, therefore there might have been bias of results.

Another study by Kadri et al. in attempt to find the more proximal segment of the STA found a surface landmark of a perpendicular line measuring 5 cm in length drawn from a point two-thirds the distance from the lateral canthus to tragus would expose the eSF. In their study, the M_4_ branch larger than 1 mm was found in 93% of the specimens.[20] However, the study used a skin marking in cadaver, thus, raising a concern during actual surgery where a skin is usually flapped to one side before opening the skull, and the marking position could be changed from the previously planned position. Pena-Tapia et al. tried to answer this question. They designed a marking template made of stiff, transparent plastic material with two intersecting lines representing the OM line and the 6 cm perpendicular line. The template was then employed together with MRI during presurgical planning.[21] This procedure did give a proper location of eSF, however, the use of template was not an affordable method in all centers.

Our study was based on this consideration. The use of bone marking is reliable and is easily employed in all centers. In this study, we found that CP was located within 1 cm above and below SF, and SS was located within 0.5 to 2 cm below SF. CP was significantly closer to SF compared with SS. A point drawn from a 6 cm perpendicular line from the OM line can be used as landmark for eSF, and a 3–4 cm craniotomy will then be able to expose the fissure. However, application of SS is also possible, especially because SS is a bony landmark and is reliably applied after skin incision, therefore it can avoid skin-marking shifting. In our series, the average distance of SS from SF was 1.5 cm below SF, and the variety of data was still in a reasonable range both on the right and left sides (Figure 5). Therefore drawing a line 1.5 cm from SS in perpendicular direction to OM line is a reliable option to find eSF.

### The course of Squamous suture and Sylvian fissure

The SF, which has a surgical importance, such as in the pterional approach, has received little attention in the neurosurgical literatures. SF is the single most identifiable feature of the superolateral face of the brain, and together with the underlying sylvian cistern it constitutes the most frequently used microneurosurgical corridor through its opening. The fissure narrows superiorly as the frontal and the temporal lobes approach each other over a length of 15–20 mm.[1] Yasargil reported that the width of the SF was about 0.5–1.0 cm on the surface. It contains the middle cerebral artery with its major branches and origins of the lenticulostriate, temporopolar and anterior temporal arteries. The superficial and deep sylvian veins are within SF as well.[22].

Anterior sylvian point (ASP), which divides SF in its main anterior and posterior rami, was considered as a good microsurgical starting point for anatomical orientation for SF opening.[8] Ribas et al. in their cadaveric study found out that ASP was located at the anterior aspect of SS just behind the pterion, was located 0.18±0.41 cm superior to anterior squamous point and 0.02±0.53 cm posterior to anterior squamous point.[8] Gibo et al. in their middle cerebral artery study found out that a distance between EAM and ASP was 47 mm.[23].

Interestingly in our present study, we found out that SF intersects with SS from 2–4 cm in front of EAM. Both results on the right and left sides did not show any significant differences. Our result confirmed the value mentioned by Ribas et al. regarding the ASP, and it gives more details concerning the course of SF along SS in actual patients. We did not perform any measurement beyond 4 cm because SS and SF were hardly visible anymore. However, since Ribas et al. did not mention any exact distance of ASP from the pterion, and another reference from Gibo et al. only mentioned rough distance from EAM to ASP of around 47 mm,[23] therefore using our result at 4 cm as an external cranial landmark for identifying the ASP is also reasonable, because we have already understood the course pattern of SF along SS from this study.

Another interesting point is a landmark for the inferior rolandic point, which corresponds to the central sulcus inferior extent projection to SF. Ribas et al. studied its position relative to the external cranial surface, and they found out that the superior squamous point was situated along the most superior segment of the SS, with an average height of 4.02±0.49 cm measured from preauricular depression point.[24] Our result showed that highest value of SS was located at 2 cm on the right side (averaged 4.92 cm) and 1.5 cm on the left side (averaged 4.97 cm) measured from a perpendicular line to OM line. The highest point of SS in our result showed no significant difference to its correspondent SF, demonstrating the start point of intersection between SF and SS. Thus, it confirmed the result mentioned by Ribas et al. where the superior squamous point was found superior to SF in 5 specimens (16%), at SF level in 20 specimen (65%), and inferior to SF in six (19%).[24]

Our clinical application also proved its efficacy. By placing the SS as the center of the craniotomy, SF was clearly viewed at the center of the operative field without any necessary advanced guidance system. eSF and its adjacent arteries were also successfully identified in all 5 patients within the distance of 1.5 cm above SS perpendicular to OM line; the recipient artery was unidentified only in one patient with a severe moya-moya disease, however, alternative recipient artery was easily found along the course of SF and bypass was successfully performed.

### Merits of bony landmark

Despite advancement in image guidance, including real time ones, not infrequently we encountered equipment malfunction or navigational shifts and/or errors. In this situation, neurosurgeon should possess the anatomic knowledge to ‘conventionally’ correct or continue the scheduled surgery. In another instance, where image guidance or real time navigation tools are not available in hospitals, neurosurgeon is expected to perform efficient surgery with whatever available tools.

In regards to simple and reliable landmarks, this is where SS comes in merits for clinical application. The surgeon can perform precise craniotomy without the necessity of using any advanced image guidance; and it is reliable since it is a bony landmark, therefore it is not shifted.

### Conclusions

The eSF represents the optimal target area on the lateral cerebral surface to expose suitable recipient vessels for STA-MCA bypass surgery, and we have learnt in this study that the simple technique described here has allowed us to identify eSF during surgery based on bone marking using SS. The course of SF along SS in actual patient's images using Osirix DICOM viewer has also been analyzed. We believe our results have added new information to neurosurgical literatures, and it can be easily applied everywhere because of its simplicity and reliability. The use of this landmark in clinical field and how it will give better orientation to operator is our next aim of study.
